# Efficient Electrochemical Sensor Based on Gold Nanoclusters/Carbon Ionic Liquid Crystal for Sensitive Determination of Neurotransmitters and Anti-Parkinson Drugs

**DOI:** 10.15171/apb.2020.006

**Published:** 2019-12-11

**Authors:** Nada Farouk Atta, Ahmed Galal, Ekram Hamdy El-Ads, Aya Essam Galal

**Affiliations:** Chemistry Department, Faculty of Science, Cairo University, 12613 Giza, Egypt.

**Keywords:** Neurotransmitters, Anti-Parkinson drugs, Gold nanoclusters, Carbon ionic liquid crystal electrode, L-dopa, Carbidopa

## Abstract

***Purpose:*** Herein we introduce a simple and sensitive sensor for the electrochemical determination of neurotransmitters compounds and anti-Parkinson drugs.

***Methods:*** The electrochemical sensor (Au/CILCE) based on gold nanoclusters modified carbon ionic liquid crystal (ILC) electrode was characterized using scanning electron microscopy and voltammetry measurements.

***Results:*** The effect of ionic liquid type in the carbon paste composite for the electro-catalytic oxidation of L-dopa was evaluated. Highest current response was obtained in case of ILC compared to other studied kinds of ionic liquids. The effective combination of gold nanoclusters and ILC resulted in extra advantages including large surface area and high ionic conductivity of the nanocomposite. L-dopa is considered one of the most important prescribed medicines for treating Parkinson’s disease. Moreover, a binary therapy using L-dopa and carbidopa proved effective and promising as it avoids the short comings of L-dopa mono-therapy for Parkinson’s patients. The Au/CILCE can detect L-dopa in human serum in the linear concentration range of 0.1 μM to 90 μM with detection and quantification limits of 4.5 nM and 15.0 nM, respectively. Also, the Au/CILCE sensor can simultaneously and sensitively detect L-dopa in the presence of carbidopa with low detection limits.

***Conclusion:*** The sensor is advantageous to be applicable for electrochemical sensing of other biologically electroactive species.

## Introduction

Neurotransmitters; chemical messengers in the synaptic transmission process, are important in maintaining human health through their role in biological, physical and pharmacological processes. Any change in the activities of these compounds may result in severe diseases like Alzheimer’s disease, schizophrenia and Parkinson’s disease. The measurement of different neurotransmitters with high sensitivity, selectivity and cost-effectiveness becomes a challenge for clinical diagnosis, medical treatment and neuroscientists. Dopamine (DA), epinephrine (EP), norepinephrine (NE), serotonin (ST) and L-dopa are important types of catecholamine neurotransmitters.^1,2^ 3,4-dihydroxy phenyl acetic acid (DOPAC) is the DA metabolite.^2^ L-dopa (3,4-dihydroxy-1-phenylalanine), a natural amino acid in humans, plants and some animals, is the biological precursor of different neurotransmitters particularly DA, EP and NE. L-dopa can be bio-synthesized in the body by the aid of L-tyrosine. Also, it can be utilized as a medication drug for the treatment of epilepsy and Parkinson’s disease.^3-9^ After taking L-dopa, it can be converted into DA in the human body through a catalytic reaction with the aid of dopa-decarboxylase enzyme and can be stored in dopaminergic neurons.^4-6^ L-dopa can be used in the healing of tremors, rigidity, spams, postural instability, movement and weak control of muscles slowness associated with Parkinson’s disease or with some drugs like perphenazine, fluphenazine and chlorpromazine.^4,6^ However, some significant side effects can be observed with the long-term administration of L-dopa like dyskinesia, paranoia schizophrenia, gastritis, arrhythmias, hair loss, nausea, hypotension, confusion and disorientation.^3,4,7,9^ On the other hand, L-dopa can be auto-oxidized resulting in toxic metabolites like quinones, semi-quinones and free radicals.^4^ Thus, there is an essential need to determine L-dopa concentration in biological fluids and pharmaceutical formulations for the aim of controlling its dosage.^3-9^ Different electrochemical approaches have been discussed for the sensitive determination of L-dopa based on carbon nanotubes,^10-12^ graphene,^13,14^ metal/metal oxides nanoparticles,^15-19^ etc.

**Scheme 1:**
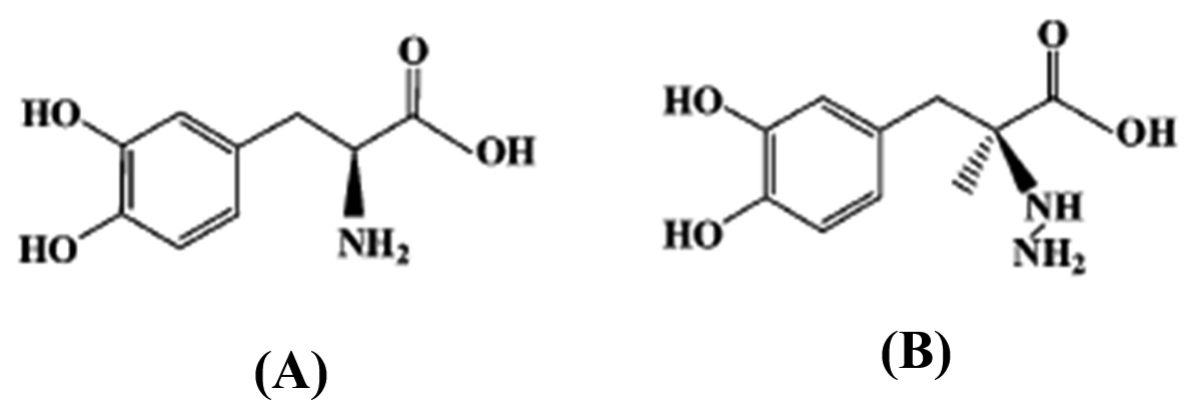
Chemical structures of **(A)** L-dopa, and **(B)** carbidopa.^20-24^

However, a therapy using L-dopa alone showed some drawbacks; namely large dosage/day, achieving the therapeutic benefits after 1-2 months, and central and peripheral side effects (rapid peripheral conversion of L-dopa to DA resulting in decreasing the therapeutic effect and causing vomiting and nausea symptoms). Therefore, a combination of carbidopa with L-dopa proved to have an active and effective role as anti-Parkinson’s drugs. L-dopa is categorized as central nervous system agent and is converted to DA in the brain. While carbidopa is categorized as decarboxylase inhibitor working by inhibiting L-dopa from breaking down before reaching the brain as it prevents the peripheral production of DA from L-dopa and therefore, L-dopa would be more rapidly and readily available for the brain metabolism. This would allow a use of lower L-dopa dosage which resulted in less vomiting and nausea symptoms.^20^ The structures of L-dopa and carbidopa were shown in Scheme 1 (A, and B).

Several modified surfaces have been mentioned in literature for the simultaneous determination of L-dopa and carbidopa based on carbon nanotubes paste electrode,^21^ graphene,^14,22^PbO_2_/carbon paste electrode (CPE),^23^ nanostructuresmodified/CPE,^24^ TiO_2_-ionic liquid/CPE,^17^ etc.

CPEs have found wide era of applications in electrochemistry due to its facility of elaboration, wide potential window and lower residual current values compared to other solid electrodes.^25^ Gold nanoparticles have emerged as microelectrodes array and may be aggregated forming clusters showing fascinating characteristics like large surface area, nano-dimensional size, good biocompatibility, high catalytic activity, improved conductivity, enhanced mass transport and distinctive optical, molecular-recognition and electronic properties. They are considered as potential candidates in different applications like microelectronics, optics, materials science, nanotechnology, catalysis, sensors, etc.^25-28^ Gold nanoparticles/reduced graphene oxide nanohybrid modified GC,^26^ gold nanoparticles modified CPE,^25,27,28^ gold nanoparticles/cysteine self-assembled monolayer modified gold electrode^29,30^ and gold nanoparticles modified carbon ionic liquid electrode^31-34^ have been utilized for the electrochemical determination of different analytes of biological interest.

Carbon ionic liquid electrode (CILE) has been employed on a large scale in the domain of electrochemical sensing of various electroactive species in different samples. This was attributed to its characteristics of high electrical conductivity, good electrochemical performance in terms of increased current response, enhanced reversibility, wide electrochemical potential window, renewable surface and fouling resistance.^31-34^ Recently, ionic liquid crystal (ILC) has arisen as an interesting compound in different fields like energy storage systems, solar cells, anisotropic ion-conductive materials, molecular electronics, electrochromic materials, synthesis approaches, universal solvents, anisotropic photo-luminescent soft materials, catalysis and sensors.^35-42^ ILC, integrated image of ionic liquid and liquid crystal, comprises of oppositely charged ions and its structure is settled through the electrostatic interactions between ions. ILC showed fascinating properties like anisotropic ionic conductivity, macroscopic orientation and charges transportation in liquid crystalline phases qualifying ILC to be a good modifier for CPE.^35-38^ Atta et al have proved the electro-catalytic activity of ILC modified CPE or GCE towards determination of some electroactive species of biological interest and catalytic conversion of methanol.^39-42^ Therefore, the combination of ILC and gold nanoclusters with their unique characteristics as modifiers for CPE is expected to offer enhanced electrochemical response towards the studied compounds.

The combining effect of these modifiers in the CPE has not yet been cited in the literature. In this paper, we introduce a simple and effective electrochemical approach for the sensitive determination of neurotransmitters andanti-Parkinson drugs. This approach is based on gold nanoclusters and ILC modified CPE “Au/CILCE”. The proposed surface exhibits enhanced electro-catalytic activity towards L-dopa determination in real samples. Besides, this sensor can simultaneously and sensitively determine L-dopa and carbidopa in human serum with low detection limits for both compounds. Also, it shows the ability for simultaneous determination of L-dopa with other common interfering species like paracetamol, uric acid (UA), ascorbic acid (AA) and ST.

## Materials and Methods

### Apparatus

The voltammetry measurements were carried out with a BAS Epsilon electrochemical analyzer in a three electrode cell. The electrodes were Ag/AgCl, a platinum wire and a CPE. Quanta 250 FEG instrument was used for scanning electron microscopy (SEM) measurements. (All the details about the electrodes and the instruments were mentioned elsewhere).^39^

### Chemicals and solutions

Graphite powder (<20 µm, synthetic), parafﬁn oil, 1-butyl-1-methyl piperidinium hexaﬂuorophosphate (ILC), 1-butyl-4-methyl pyridinium tetraﬂuoroborate (IL1) and 1-ethyl-3-methyl imidazolium-1,1,2,2-tetra-ﬂuoroethanesulfonate (IL2) ionic liquids, hydrogen tetra-chloroaurate (HAuCl_4_), KNO_3_, L-dopa, carbidopa, DOPAC, EP, NE, ST, DA, UA, AA and paracetamol (APAP) were supplied by Aldrich Chem. Co. (Milwaukee, WI. USA). The supporting electrolyte used in the present study was phosphate buffer solution (PBS was prepared from 1.0 M K_2_HPO_4_ and 1.0 M KH_2_PO_4_) of pH (2-11).

### Construction of working electrodes

**Scheme 2:**
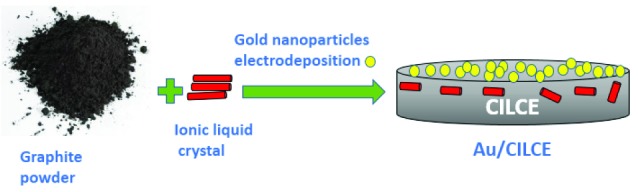
Structural representation of Au/CILCE.

Bare CPE was prepared by the same way as mentioned elsewhere.^39^ ILC modified CPE was prepared by mixing 0.3 g of ILC and 1.5 g of graphite powder with 0.5 mL of parafﬁn oil.^39^ The resulting modified electrode was represented as CILCE. Electrodeposition of gold nanoclusters on CILCE was carried out in a solution containing 6 mM HAuCl_4_/0.1M KNO_3_ under potential control at ‒ 400 mV for 400 seconds.^29,30^ The resulting electrode was represented as Au/CILCE and its structural representation was shown in Scheme 2. To evaluate the impact of ILC as a modifier of CPE, two other ionic liquids were used (IL1 and IL2). CPE modified with ionic liquid was prepared by mixing IL with graphite powder with a ratio of 1% (w/w). Then gold nanoclusters were electrodeposited as previously mentioned procedure. The resulting electrodes were represented as Au/CIL1E and Au/CIL2E, respectively.

## Results and Discussion

### Morphology study

**Figure 1:**
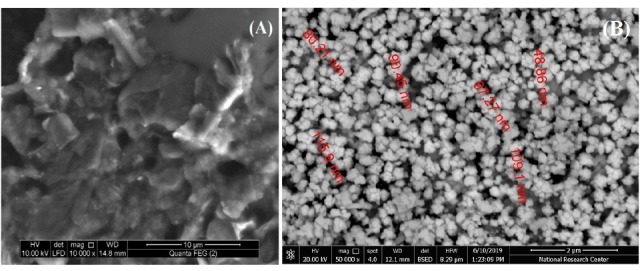
Scanning Electron Micrographs (SEM) of (A) Carbon ionic liquid crystal electrode (CILCE) and (B) Gold carbon ionic liquid crystal electrode (Au/CILCE).

The morphology of the modified electrode strongly affects its response and the electro-catalytic activity towards the studied compounds. As mentioned elsewhere, bare CPE showed random graphite flakes with low conductivity attributing to the existence of paraffin oil (Figure not shown). Figure 1(A and B) shows the SEM of CILCE and Au/CILCE, respectively. The SEM of CILCE showed a muzzy shape where ILC distributed between the graphite flakes due to its mild viscosity (Figure 1A). ILC manipulated the conductivity level of bare CPE and may lead to more ordered surface for the electrodeposited gold nanoclusters.^39^ The SEM images of Au/CILCE showed well-ordered and homogenously distributed gold nano-clusters (Figure 1B).^43^ The cluster size is in the range of (48.9-115.9 nm).

Gold nanoclusters influence the surface area of CILCE surface and improve the surface conductivity. The electrochemistry of 1 mM Fe(CN)_6_-/Fe(CN)_6_- in 0.1 M KCl was investigated^44^ at different surfaces; CPE, CILCE and Au/CILCE (Figure not shown) and the active surface areas were calculated using Randles-Sevcik equation. The calculated active surface area in case of Au/CILCE (0.7578 cm^2^) was higher than that in case of CILCE (0.5008 cm^2^) and bare CPE (0.1987 cm^2^). These results indicated the enhancement in the active surface area upon the inclusion of ILC inside the CPE and further electrodeposition of gold nanoclusters.

### Electrochemical behavior of different neurotransmitters at Au/CILCE

**Figure 2:**
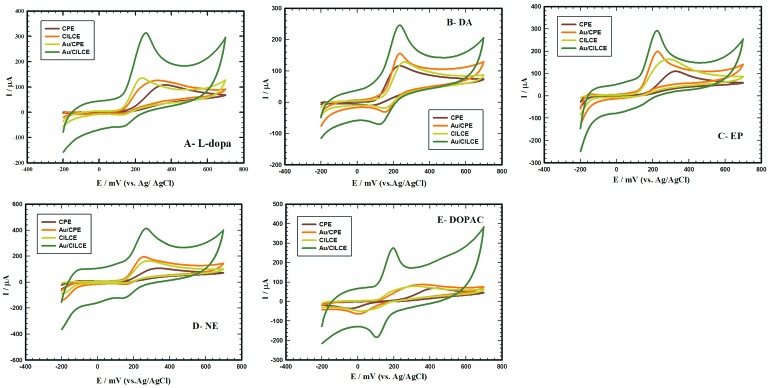
Cyclic voltammograms of 1 mM of **(A)** L-dopa, **(B)** dopamine (DA), **(C)** epinephrine (EP), **(D)** norepinephrine (NE) and **(E)** DOPAC each prepared in 0.1 M PBS/pH 7.4 recorded at different working electrodes; bare carbon paste electrode (CPE), carbon ionic liquid crystal electrode (CILCE), gold carbon paste electrode (Au/CPE) and gold carbon ionic liquid crystal electrode (Au/CILCE), scan rate 50 mV s^-1^.

The effective combination of ILC and gold nanoclusters and its influence on the electrochemical behaviors of different studied compounds (L-dopa, DA, EP, NE and DOPAC) was illustrated at different surfaces. Figure 2 (A, B, C, D and E) displays the cyclic voltammograms of 1 mM/0.1 M PBS/pH 7.4 of L-dopa, DA, EP, NE and DOPAC at different surfaces (CPE, CILCE, Au/CPE and Au/CILCE), respectively. The characteristic electrochemical data including the anodic peak current and anodic peak potential values of all the studied compounds were summarized in (Table 1). Upon modification of CPE with ILC or gold nanoclusters, a slight increase in the anodic peak current was observed in all the studied compounds. The synergetic combination of ILC and gold nanoclusters as modifiers for CPE resulted in a remarkable increase in the anodic and cathodic peak current values and shift of the anodic peak potential to more negative values in all the studied compounds. Also, in some cases (DOPAC and DA) there was an obvious decrease in the potential peak difference and an enhancement in the reversibility at Au/CILCE. These observations reflected the facilitation of electron transfer process at Au/CILCE in all cases suggesting the electro-catalytic activity of the proposed surface. Gold nanoclusters offered unique electronic properties, large surface to volume ratio, electrical conductivity and improved surface area.^44^ ILC offered enhanced ionic conductivity and molecular orientation ordering. So, the influential integration of ILC and gold nanoclusters in CPE resulted in enhancement of the charge transfer between the electroactive species and the electrode surface and improved the analytical performance of the sensor as will be discussed in the upcoming sections.

**Table 1:** Summary of CV results obtained at carbon paste electrode (CPE), carbon ionic liquid crystal electrode (CILCE), gold carbon paste electrode (Au/CPE) and gold carbon ionic liquid crystal electrode (Au/CILCE) for 1 mM of each compound in 0.1 M PBS/pH 7.40, scan rate 50 mV s^−1^

**Compound**	**CPE**			**CILCE**			**Au/CPE**			**Au/CILCE**		
	**E**_pa_ **mV**	**I**_pa_ **µA**	**Current increase** ^*^	**E**_pa_ **mV**	**I**_pa_ **µA**	**Current increase** ^*^	**E**_pa_ **mV**	**I**_pa_ **µA**	**Current increase** ^*^	**E**_pa_ **mV**	**I**_pa_ **µA**	**Current increase** ^*^
L-dopa	**363**	**107**	**-**	**318**	**123**	**1.15**	**235**	**130**	**1.21**	**260**	**265**	**2.48**
EP	326	110	-	285	160	1.45	226	195	1.77	223	230	2.09
NE	337	100	-	281	160	1.6	257	180	1.8	270	300	3.00
DA	235	116	-	260	123	1.06	236	144	1.24	234	188	1.62
DOPAC	423	67	-	316	74	1.10	350	80	1.19	196	190	2.84

^*^ Current increase normalized to bare CPE, EP; epinephrine, NE; norepinephrine, DA; dopamine.

### Influence of type of ionic liquid on the electrochemical signal of L-dopa

**Figure 3:**
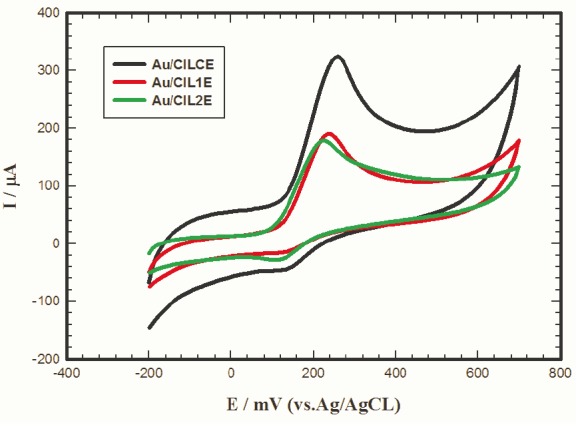
Cyclic voltammograms of 1 mM L-dopa/0.1 M PBS/pH 7.4 recorded at different working electrodes; gold carbon ionic liquid crystal electrode (Au/CILCE), gold carbon ionic liquid 1 electrode (Au/CIL1E) and gold carbon ionic liquid 2 electrode (Au/CIL2E), scan rate 50 mV s^-1^.

ILC has interesting properties like high ionic conductivity and good charges transportation in liquid crystalline phases compared to room temperature ionic liquids, qualifying ILC to be a good modifier for CPE.^35-38^ Therefore, it was necessary to investigate the electrochemical response of 1 mM L-dopa/0.1 M PBS/pH 7.4 at Au/CILCE, Au/CIL1E and Au/CIL2E as shown in Figure 3. Higher current response of L-dopa was obtained at Au/CILCE (265 µA) compared to Au/CIL1E (140 µA) and Au/CIL2E (135 µA) emphasizing the good catalytic properties of the ILC modified nanocomposite.

### Influence of scan rate on the electro-oxidation of L-dopa

The influence of scan rate on the oxidation peak current of L-dopa at the Au/CILCE was studied using CV mode. (Figure S1; Inset) showed CVs of 1 mmol L^−1^ L-dopa/0.1 M PBS/pH 7.4 using the modified electrode at different scan rates from 10 to 400 mVs^−1^. The results indicated that the relation between peak current and the square root of scan rate was linear in the studied range (Figure S1, Supplementary file 1) according to equation (1):

I_p_ (µA) = 765.2 *v*^1/2^ (V/s)^1/2^ + 70.93 (1)

correlation coefficient R^2^ = 0.994.

This linear relation reflects that the electro-oxidation of L-dopa at Au/CILCE is under diffusion control.^2-7^ Diffusion coefficient “D, cm^2^ s^-1^” values were calculated from Randles-Sevcik equation for a quasi-reversible process.^45-48^ For the electro-oxidation process of L-dopa where n = 2, C =1×10^-6^ mol/cm^3^, and the geometrical surface area of CPE (A) = 0.312 cm^2^; the calculated D values of L-dopa at CPE, CILCE, Au/CPE and Au/CILCE were 4.19×10^-6^cm^2^ s^-1^, 5.53×10^-6^cm^2^ s^-1^, 6.18×10^-6^cm^2^ s^-1^ and 2.47×10^-5^cm^2^ s^-1^, respectively displaying the highest value at Au/CILCE. This suggests that there is enhancing in the mass transfer process of L-dopa from solution bulk to electrode surface and/or the charge transfer process during the electro-oxidation of L-dopa at electrode surface.

### Influence of supporting electrolyte pH on the electro-oxidation of neurotransmitters

The influence of pH value of the supporting electrolyte on the current response of different studied compounds (L-dopa, DOPAC, NE, EP and DA) was investigated and shown in Figure S2A. Inset of Figure S2B showed the cyclic voltammograms of 1 mM L-dopa/0.1 M PBS of different pH values in the range of 2.5-11 at Au/CILCE. The supporting electrolyte pH influenced both of the anodic peak current and anodic peak potential values of L-dopa. As shown in Figure S2B, the anodic peak potential of L-dopa was shifted to negative potential with increasing the pH value because of L-dopa deprotonation during the oxidation. This demonstrated that the electron transfer process of L-dopa includes protonation/deprotonation steps.^2,3,11,12,15^ Figure S2B showed a linear relationship between the peak potential (Ep) and the pH (2.5⟶11) with a slope of ˗ 56.0 mV/pH ~ close to Nernstian slope, which expressed by equation (2):

E_pa_ (V) = 0.665 – 0.056 pH (2)

(R^2^ = 0.994)

This manifests that the electro-oxidation of L-dopa at Au/CILCE proceeds through two electrons/two protons which is in agreement with the literature.^2,3,11,12,15^ Also, the inset of Figure S2B showed the influence of pH on the anodic peak current of L-dopa displaying the maximum current value at pH 7.4.Therefore the present study was performed at pH 7.4.

### Stability, precision and robustness

Stability of the electrochemical signal of the studied analyte is an important parameter reflecting the characteristics of the proposed sensor. Figure S3 investigated the stability of the electrochemical signal of 1 mM L-dopa/0.1 M PBS/pH 7.4 at Au/CILCE up to 25 cycles. A good stable response in terms of current and potential values was obtained for L-dopa at Au/CILCE manifesting the antifouling characteristics of the proposed surface. The current response retained 96% of its initial value upon repeating cycle up to 25 cycles.

Reproducibility of the present method was checked via intra-day and inter-day precisions. The experiments were studied by analytical testing the same sensor four times in the same solution with the same concentration or using four similar sensors in four separate runs (four times), respectively. The calculated values of relative standard deviation (RSD) for L-dopa were low; (0.96%) and (1.18%) for intra-day and inter-day precisions, respectively.^2,40^ These small values manifested the good precision and stability of the electrochemical response at this surface. Furthermore, the influence of slight variation in the experimental conditions on the stability of the current response of oxidation of L-dopa at the Au/CILCE was examined to check the robustness of the proposed method. The parameters under examination were deposition time of gold nanoclusters (400 s ± 20 s), amount of ILC (0.5 g ± 0.001 g) and pH of PBS (7.4 ± 0.1). Small RSDs of 2.08%, 1.29% and 1.79%, respectively were realized asserting the trustiness and robustness of the suggested method during the quantitative analysis of L-dopa under the experimental conditions.

### Electrochemical determination of L-dopa in human serum at Au/CILCE

**Figure 4:**
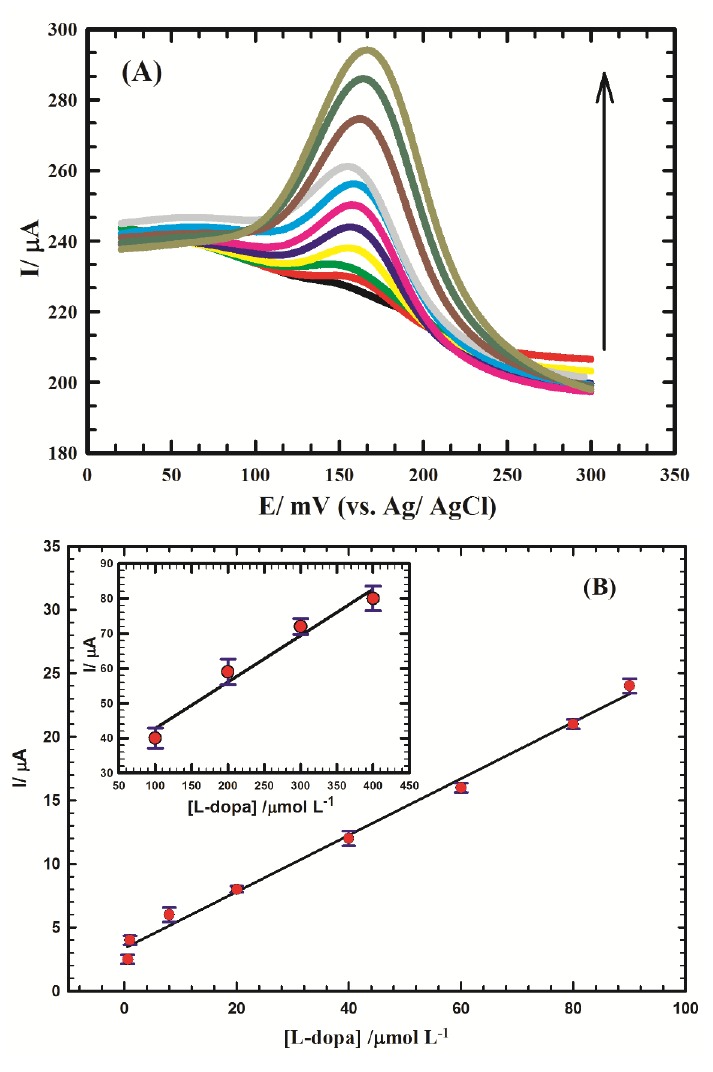
**(A)** DPVs of gold carbon ionic liquid crystal electrode (Au/CILCE) with successive additions of L-dopa from 0.1 μM to 400 μM. **(B)** Calibration curve of the smaller concentration range (0.1 µMto 90 µM) of L-dopa/0.1 M PBS/pH 7.4. **Inset:** Calibration curve of the larger concentration range (100 µMto 400 µM) of L-dopa/0.1 M PBS/pH 7.4.

Quantitative assay of L-dopa/0.1 M PBS/pH 7.4 in human serum as a real sample was achieved at Au/CILCE sensor by differential pulse voltammetry (DPV) mode. The proposed method was validated according to WHO guidelines^49^ with respect to linearity, limit of detection and limit of quantification. Standard additions were applied, and the sample was diluted with PBS/(pH 7.4) 20 times to decrease the effect of matrix complexity. The peak current response of L-dopa increases with increasing the concentration in the range of (0.1 to 400 μM) for L-dopa as shown in (Figure 4A). The peak current (Ip_a_) was found to be linearly dependent on the concentration in ranges of 0.1 μM to 90 μM and 100 µM to 400 µM, respectively as illustrated in (Figure 4B and the inset), and represented by equations (3, 4):

Ip (μA) = 0.223 C (μM) + 3.35 (3)

(R^2^= 0.993).

The analytical performance of the proposed surface can be identified in terms of sensitivity of 0.223 μA/μM, detection limit (DL) of 4.5 nM and quantification limit (QL) of 15.0 nM.

Ip (μA) = 0.133 C (μM) + 29.5 (4)

(R^2^= 0.967).

The equations used for the calculation of limits of detection and quantification were described elsewhere.^34-36^

It is crucial to compare the performance of the proposed nanocomposite with that mentioned in literature to evaluate its response and confirm its characteristics. A comparison of DL, sensitivity and linear dynamic range was summarized in Table 2. The proposed nanocomposite showed good analytical performance compared to other surfaces, besides its ease of fabrication and cost-effectiveness. ILC played a major role in increasing the conductivity of the composite and enhancing the kinetic process compared to other composites modified with IL as shown in Table 2. Furthermore, this reflected the synergistic interaction of the individual components of the proposed nano-composite which was attributed to their intrinsic catalytic characteristics.

**Table 2:** Comparison of figures of merit for gold carbon ionic liquid crystal electrode (Au/CILCE) with different modified electrodes mentioned in literature for L-dopa determination

**Electrode**	**Linear dynamic range (μM)**	**Sensitivity (μA/μM)**	**Detection limit (nM)**
Gold and palladium nanoparticles modified nano-porous stainless steel^3^	5-55	0.1518	200
Electrochemically reduced graphene oxide/poly-glycine composite modified glassy carbon electrode^4^	1-50	1.01	150
NiO nanoparticle-ionic liquid modified CPE^6^	0.7–900	0.0045	400
Reactive blue 19 and multi-walled carbon nanotube modified glassy carbon electrode^8^	1.37-92.59	Not reported	370
Carbon nanotubes paste electrode modified with iron-phthalocyanines^11^	10-80	0.0745	504
CILE modified with multi-walled carbon nanotubes and cobalt hydroxide nanoparticles^12^	0.1–300	1.59	75
Graphene nanosheets modified glassy carbon electrode^14^	1-16	2.15	800
Gold nanoparticles functionalized 8-hydroxyquinoline modified glassy carbon electrode^16^	3.0–10	Not reported	310
Au/CILCE (This work)	0.1-90	0.223	4.5

### Applicability in real serum sample

To investigate whether the proposed sensor is reliable and promising for the determination of L-dopa in serum samples, we spiked various concentrations of L-dopa to serum samples (diluted 20 times with PBS as mentioned before) and determined L-dopa spiked in serum samples. As presented in Table S1 (Supplementary file 1), the recovery values were in the range of 96.43% to 101.52% for L-dopa with RSDs in the range of 1.13% to 6.99% for L-dopa. Thus, the sensor; Au/CILCE has good accuracy for detection of L-dopa in serum samples.

The results were in good agreement with those obtained using other reported methods showing that this method achieved the validation for L-dopa quality control analysis.^49^ The summary of recoveries and RSD values were displayed in Table S1.The above results confirmed the practicality of using the Au/CILCE for the determination of L-dopa in human serum.

### Analysis of L-dopa and carbidopa in their pharmaceutical tablets

In the following experiments,we examine any interference from the compounds that could be encountered from tablet excipients and affect the current response of the drug. The two medicines namely Larodopa containing (100 mg L-dopa/tablet) and carbidopa containing (25 mg carbidopa/tablet) were investigated at the Au/CILCE. The procedure was run as follows: First the tablet was ground and stoichiometrically dissolved in PBS (pH = 7.4). Then the solution was sonicated and filtered off any non-dissolved residue. From each tablet stock solution, measured volumes were transferred to the electrochemical cell. The concentration ranges (8 μM−100 μM), and (20 µM−100 µM) were used for recovery tests of L-dopa and carbidopa respectively using DPV mode. Known standard amount of each drug (2 µM) was added to the pre-analyzed formulations of L-dopa and carbidopa separately before recovery studies were carried out. Recovery tests were repeated three times for each concentration. Table S2 (A & B) showed the recovery and RSD values. The data illustrated a good exactness of the proposed method.

### Effect of common interferences on the electrochemical current signal of L-dopa

#### Determination of L-dopa in presence of common interferences

**Figure 5:**
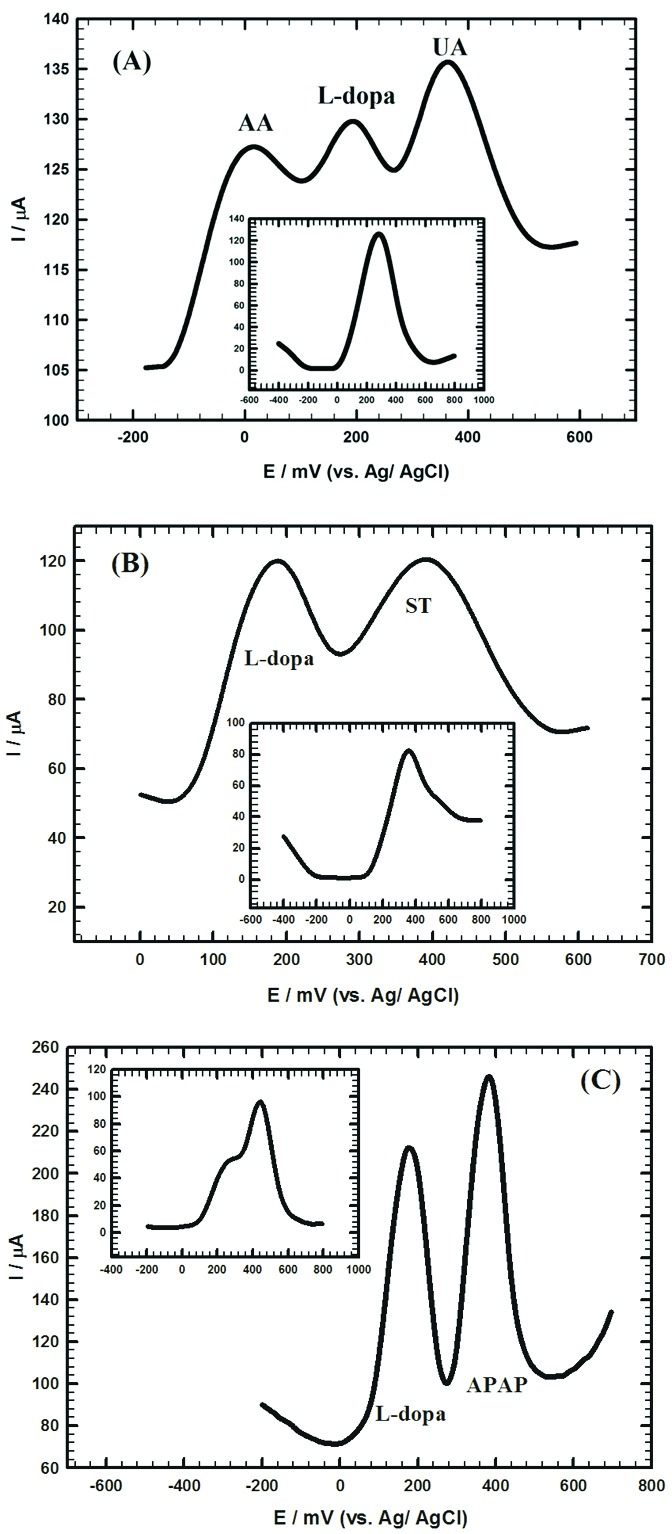
(**A**) DPV for L-dopa (200 µM), ascorbic acid (AA) (3 mM) and uric acid (UA) (0.3 mM) mixture at gold carbon ionic liquid crystal electrode (Au/CILCE). **Inset:** DPV for the same mixture at carbon paste electrode. (**B**) DPV for 1 mM L-dopa and 1 mM serotonin (ST) at gold carbon ionic liquid crystal electrode (Au/CILCE). **Inset:**DPV for the same mixture at carbon paste electrode. (**C**) DPV for 1 mM L-dopa and 1 mM paracetamol (APAP) at gold carbon ionic liquid crystal electrode (Au/CILCE). **Inset:**DPV for the same mixture at carbon paste electrode.

The influence of various interferences on the electrochemical signal of L-dopa is very crucial from the medical point of view. AA and UA are important compounds in the living systems and they coexist in biological fluids like blood or urine. L-dopa administration may cause the elevation of UA concentration by the prohibition of its renal secretion.^8^ Figure 5A and inset showed the DPVs of 0.2 mM L-dopa in presence of 3 mM AA and 0.3 mM UA prepared in 0.1 M PBS/pH 7.40 at Au/CILCE and bare CPE, respectively. An overlapped combined peak was obtained at bare CPE while three well-separated peaks were obtained at 64 mV, 240 mV and 396 mV for AA, L-dopa and UA at Au/CILCE, respectively. On the other hand, L-dopa exerts direct effects on the ST levels because ST neurons can decarboxylate L-dopa into DA. Therefore, simultaneous determination of L-dopa and ST is crucial.^9^ Figure 5B and inset showed DPVs of 1 mM L-dopa and 1 mM ST prepared in 0.1 M PBS/pH 7.40 at Au/CILCE and bare CPE, respectively. Two well-resolved peaks were obtained at 188 mV and 388 mV for L-dopa and ST, respectively at Au/CILCE compared to a combined unresolved peak at bare CPE. Also, patients with Parkinson’s disease use APAP, some drugs and opiates for the therapy of neuropathic pain. APAP is considered as fever reducer and pain reliever drug.^7^ Therefore, simultaneous determination of L-dopa and APAP is crucial for people under treatment by these medications.^7^ Figure 5C and inset showed DPVs of 1 mM L-dopa and 1 mM APAP prepared in 0.1 M PBS/pH 7.40 at Au/CILCE and bare CPE, respectively. Two well-resolved peaks were obtained at 144 mV and 408 mV for L-dopa and APAP, respectively at Au/CILCE compared to a combined peak at bare CPE. The previous results clearly proved the ability of the proposed surface for the electrochemical determination of L-dopa in the presence of common interferences with good potential peak separation and high current response.

### Simultaneous determination of L-dopa and carbidopa in human serum

**Figure 6:**
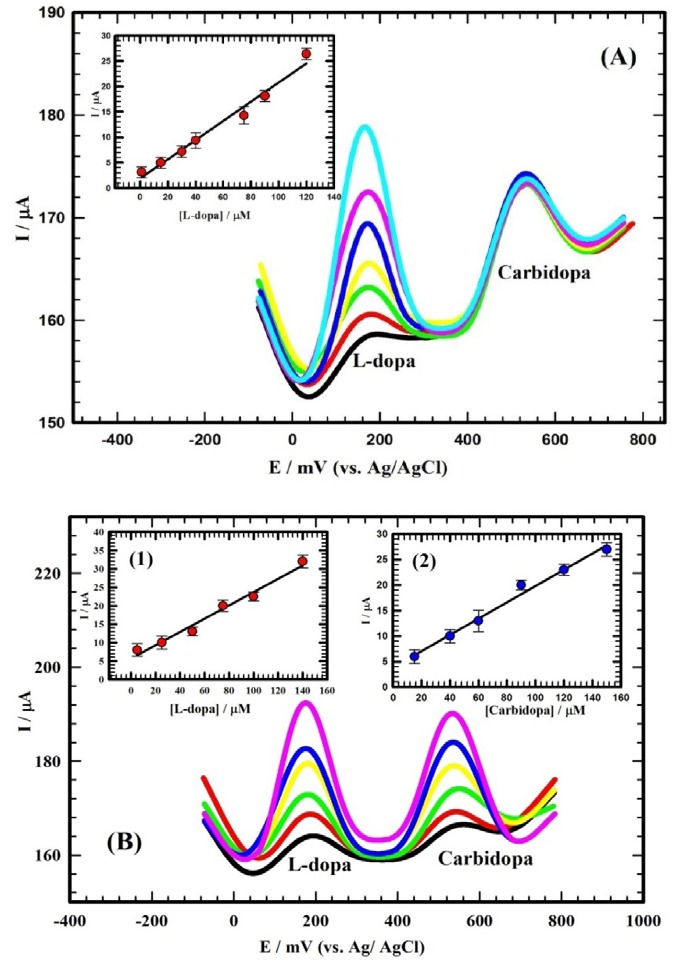
(**A**) DPVs for L-dopa in the concentration range of (1 μM - 120 μM) in presence of 60 μM carbidopa/diluted serum at gold carbon ionic liquid crystal electrode (Au/CILCE). **Inset:** Calibration curve for L-dopa in the linear dynamic range of (1 μM - 120 μM) in presence of 60 μM of carbidopa. (**B**) DPVs for L-dopa and carbidopa in the concentration ranges of (5 μM -140 μM) and (15 μM - 150 μM) respectively, in diluted serum at Au/CILCE. **Insets**(**1, 2**): Calibration curves for L-dopa and carbidopa in the linear dynamic ranges of (5 μM - 140 μM) and (15 μM - 150 μM), respectively.

A binary therapy using L-dopa and carbidopa proved effective and promising as it avoids the short comings of L-dopa mono-therapy for Parkinson’s patients. Thus, the better effective medication for Parkinson’s patients is the binary therapy of L-dopa and carbidopa. Standard additions were performed in diluted serum as described before using standard stock solution of the studied compounds. Figure 6A depicted the DPVs forL-dopa and carbidopa with varying the concentration of L-dopa (1 ⟶ 120 μM) while keeping the concentration of carbidopa constant at 60 µM using Au/CILCE. The inset of Figure 6A depicted the linear relation between the oxidation peak current and the variation in concentration of L-dopa. The following equation (5) represented this relation.

I_p_ (µA) = 0.189 C (µM) + 1.91 (5)

(R^2^= 0.980).

Figures of merit were as follows: sensitivity of 0.189 μA/μM; DL of 25.5 nMand QL of 85.0 nM.

Simultaneous determination of L-dopa and carbidopa in their mixture using Au/CILCE was not cited in the literature. Figure 6B showed the DPVs for simultaneous determination of L-dopa and carbidopa. Two distinct oxidation peaks were identified for a binary mixture of L-dopa and carbidopa. The corresponding concentrations were varied as follows: L-dopa (5 μM to 140 µM) and carbidopa (15 μM to 150 µM). The insets of Figure 6B depicted the linear relations between the oxidation peak currents and the variation in concentrations of L-dopa and carbidopa. The following equations (6, 7) represented these relations.

I_p_ (µA) = 0.185 C (µM) + 7.40 (6)

(R^2^= 0.977).

Figures of merit were as follows: sensitivity of 0.185 μA/μM; DL of 26.3 nMand QL of 87.7 nM.

I_p_ (µA) = 0.182 C (µM) + 2.59 (7)

(R^2^= 0.997).

Figures of merit were sensitivity of 0.182 μA/μM; DL of 28.1 nMand QL of 93.7 nM.

According to the above results, it was observed that the sensitivity of the proposed surface towards L-dopa in presence of carbidopa (0.189 μA/μM) was close to the sensitivity in its absence (0.223 μA/μM) in the lower concentration range. This ascertained that the electrochemical oxidation behavior of these compounds using the sensor; Au/CILCE was independent.

## Conclusion

In this work, we presented a simple and sensitive electrochemical voltammetry approach for determination of neurotransmitters compounds and anti-Parkinson drugs. The Fabrication of the sensor was based on gold nanoclusters modified carbon ILC electrode; (Au/CILCE). The combination of ILC and gold nanoclusters with distinctive properties resulted in enhancing the electrochemical responses and the charge transfer kinetics between the studied compounds and the proposed electrode. The proposed surface can sensitively detect L-dopa in the linear dynamic range of 0.1 μM to 90 μM with DL of 4.5 nM and QL of 15.0 nM. Moreover, the sensor can simultaneously and sensitively determine L-dopa and carbidopa in human serum with low detection limits of 26.3 nMand 28.1 nMrespectively. Also, simultaneous determination of L-dopa with interfering species as ST; AA and UA; and paracetamol was successfully achieved with good potential peak separation values.

## Ethical Issues

Not applicable.

## Conflict of Interest

There is no conflict of interest to declare.

## Acknowledgments

The authors appreciated the financial support from Cairo University through the Office of the President for Research Funds.

## Supplementary Materials


Supplementary file 1 contains Figures S1-S3 and Tables S1-S2.
