# Supplementary material for: Efficient Electrochemical Sensor Based on Gold Nanoclusters/Carbon Ionic Liquid Crystal for Sensitive Determination of Neurotransmitters and Anti-Parkinson Drugs
Source: Adv Pharm Bull. 2019 Dec 11;10(1):46–55. doi: 10.15171/apb.2020.006 (PMC6983987; doi:10.15171/apb.2020.006)
Supplement: Supplementary file 1 — contains Figures S1-S3 and Tables S1-S2. [file apb-10-46-s001.pdf]

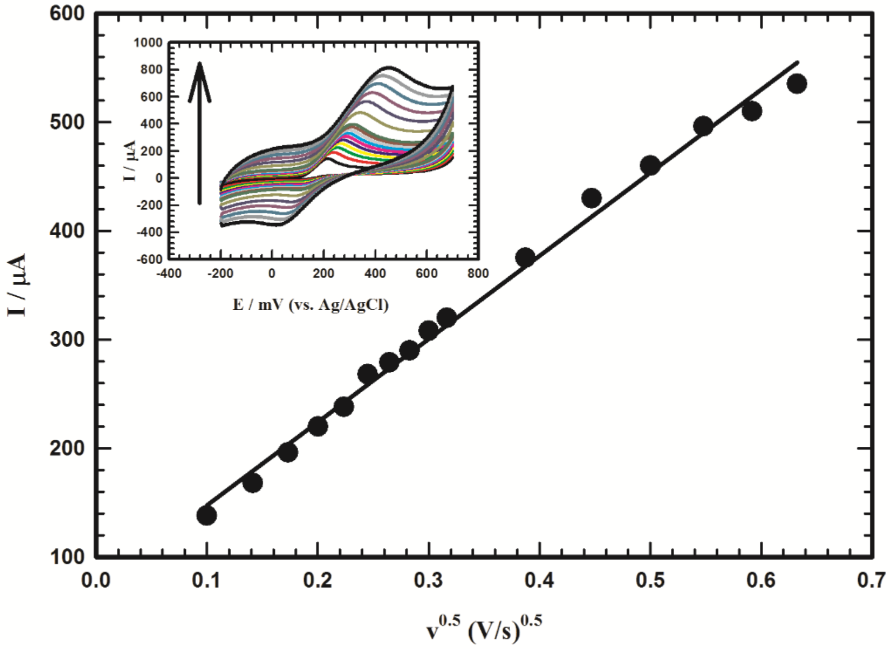

Figure S1. Plot of the anodic peak current of 1 mM L-dopa/0.1 M PBS/pH 7.4 at gold carbon ionic liquid crystal electrode (Au/CILCE) as a function of the square root of scan rate (from 10 to 400 mV/s). **Inset.** Cyclic voltammograms of 1 mM L-dopa/0.1 M PBS/pH 7.4 recorded at gold carbon ionic liquid crystal electrode (Au/CILCE) at different scan rate values from (10 to 400 mV/s).

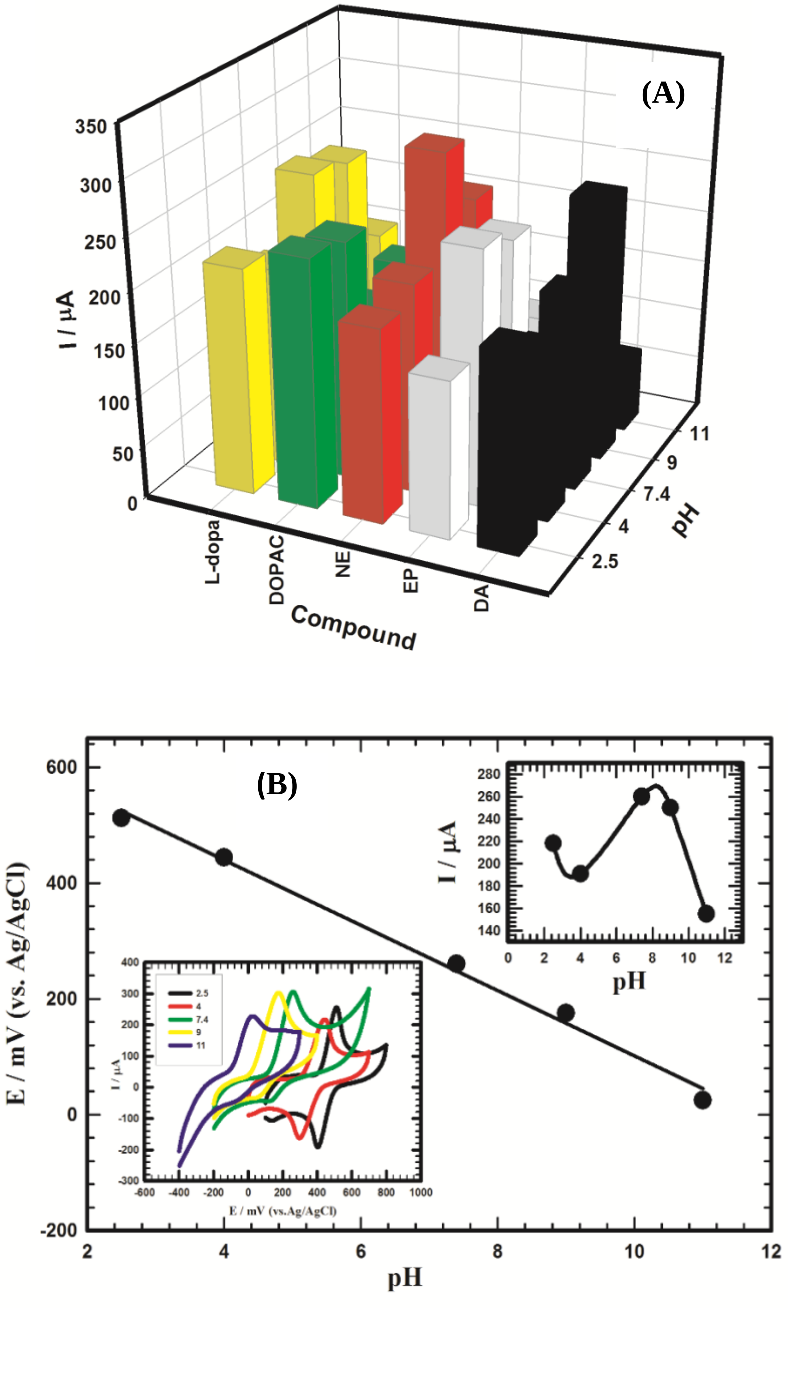

Figure S2. (A) A graph of current responses of 1 mM/0.1 M PBS of each compound; L-dopa, DOPAC, NE, EP and DA at different pH values (2.5-11) at gold carbon ionic liquid crystal electrode (Au/CILCE).

(B) Plot of the anodic peak potential of L-dopa versus the pH values. Insets: Cyclic voltammograms of 1 mM L-dopa/0.1 M PBS of different pH values from (2.5 to 11) at gold carbon ionic liquid crystal electrode (Au/CILCE) and a plot of the anodic peak current of L-dopa versus the pH values.

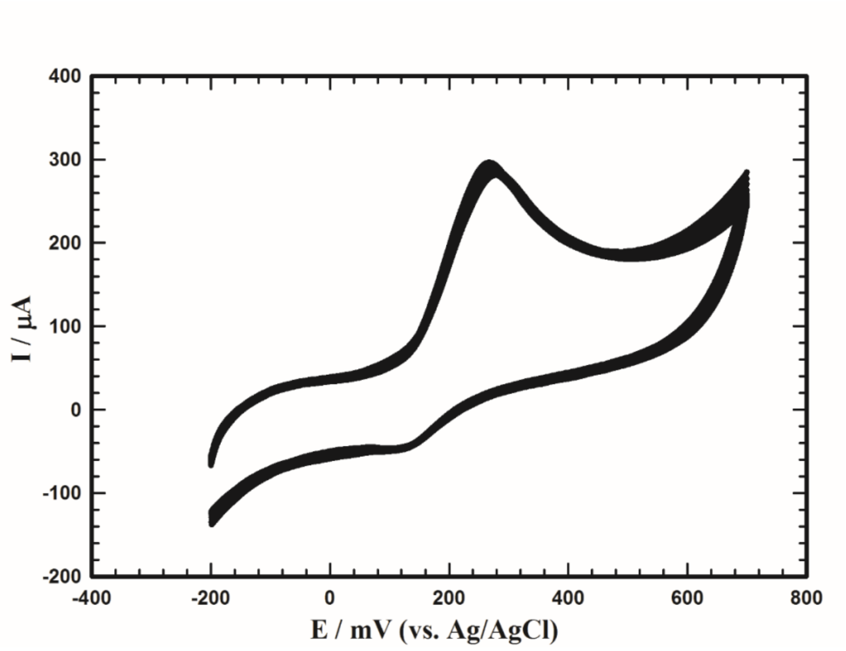

Figure S3. Repeated cyclic voltammograms of 1 mM L-dopa/0.1 M PBS/pH 7.4 at gold carbon ionic liquid crystal electrode (Au/CILCE) up to 25 cycles, scan rate 50 mV s^{-1}.

Table S1. Evaluation of the accuracy and precision of the proposed method for determination of L-dopa in serum sample.

**Sample**	**[L-dopa]/µM**	**Found/µM**	**Recovery %**
**1**	6	6.091	101.52
**2**	40	39.29	98.21
**3**	60	59.03	98.39
**4**	80	77.14	96.43
**5**	100	101.28	101.28

Table S2. Recovery data obtained by standard addition method for **(A) L-dopa, and (B) Carbidopa** in their drug formulations.

(A)

**Tablet taken (μM)[a]**	**Standard added (μM)**	**Found (μM)[b]**	**Recovery (%)**	**SD[c] × 10^{-7}**	**RSD[d] (%)**
8	2.00	10.20	102.0	4.40	6.99
20	2.00	21.93	99.67	1.42	1.89
40	2.00	42.67	101.6	3.46	2.77
60	2.00	61.85	99.76	1.53	1.13
80	2.00	81.52	99.41	2.65	1.58
100	2.00	98.38	96.45	1.29	4.37

[a]: Aliquots of the L-dopa tablet sample were injected by a micropipette with concentrations of 8–100 μM.
[b]: The represented data were an average of three determinations.
[c]: Standard deviation.
[d]: Relative standard deviation.

(B)

**Tablet taken (μM)[a]**	**Standard added (μM)**	**Found (μM)[b]**	**Recovery (%)**	**SD[c] × 10^{-7}**	**RSD[d] (%)**
20	2.00	21.93	99.68	0.354	0.500
40	2.00	41.64	99.14	6.36	5.26
60	2.00	62.00	100.00	1.41	0.930
80	2.00	82.50	100.61	2.83	1.57
100	2.00	101.3	99.31	2.12	1.06

[a]: Aliquots of the carbidopa tablet sample were injected by a micropipette with concentrations of 20–100 μM.
[b]: The represented data were an average of three determinations.
[c]: Standard deviation.
[d]: Relative standard deviation.
